# Ion-exchange resin anticoagulation: experimental data of a novel extracorporeal regional anticoagulation technique

**DOI:** 10.1186/2197-425X-3-S1-A807

**Published:** 2015-10-01

**Authors:** A Zanella, V Scaravilli, M Giani, F Magni, D Ceriani, S Sosio, S Spina, C Ferrari, S Colombo, A Pesenti

**Affiliations:** Università degli Studi di Milano Bicocca, Monza, Italy; Ospedale San Gerardo, Monza, Italy

## Introduction

Regional anticoagulation has been introduced to avoid the complications of systemic anticoagulation. The most diffuse technique for regional anticoagulation is the infusion of citrate, which exerts its anticoagulative effect by chelating calcium, an essential factor of coagulation. Despite being a “regional” anticoagulation technique, a major fraction of the infused citrate reaches the patient systemic circulation and may cause toxicity. For this reason this technique can be applied only to extracorporeal blood flows lower than ~150-180 ml/min, to limit the amount of infused citrate. Ion exchange resins are polymers which contain acidic or basic functional groups able to exchange counter-ions. We have developed a regional anticoagulation technique featuring a cation exchange resin, which exchanges calcium with sodium and thus reduces plasmatic calcium levels.

## Objectives

To investigate the feasibility and efficacy of an innovative regional blood anticoagulation technique based on cation exchange resins.

## Methods

Eight healthy swine (48.5 ± 4.5 kg) were sedated, intubated, mechanically ventilated and connected to a custom-made veno-venous extracorporeal system featuring a cartridge containing a cation-exchange resin. Blood flow was set at 150 ml/min. Systemic anticoagulation with unfractioned heparin was started and maintained for the whole experiment. During the experiment blood was sampled along the extracorporeal system before and after the resin anticoagulation treatment to measure ionized calcium and to perform thromboelastography (TEG). The heparinase-kaolin thromboelastography (HK-TEG) technique was used to evaluate coagulation, regardless of the level of systemic anticoagulation. Systemic ionized calcium and sodium levels were maintained stable by infusing a custom-made sodium-free calcium replacement solution. Three samples were withdrawn after 2, 4 and 6 hours of ion-exchange resin anticoagulation treatment.

## Results

Table 1 shows the ionized calcium levels and the TEG parameters of blood along the extracorporeal circuit, before (Blood IN) and after (Blood OUT) the ion-exchange resin anticoagulation treatment. The treatment was able to reduce ionized calcium levels to 0.25 ± 0.03 mmol/L, resulting in no sign of clot formation in samples withdrawn from the blood outlet. Systemic electrolytes were stable during the whole experiment. No adverse event ascribable to the treatment was recorded.

**Table 1:** Table 1

	Blood IN	Blood OUT	p-value
Ionized Calcium (mmol/L)	1.29 ± 0.06	0.25 ± 0.03	< 0.001
HK-TEG - R (sec)	6.8 ± 2.2	> 60	< 0.001
HK-TEG - α Angle (°)	2.2 ± 0.7	0 ± 0	< 0.001
HK-TEG - K (min)	62 ± 7	0 ± 0	< 0.001
HK-TEG - MA (mm)	66 ± 5	0 ± 0	< 0.001

## Conclusions

The ion-exchange resin anticoagulation technique proved to be effective to anticoagulate extracorporeal blood. Future experimental studies are required to evaluate the long-term feasibility and safety of the technique. This technique can potentially be applied to a higher range of blood flows, extending the applicability of regional anticoagulation to other extracorporeal treatment, such as extracorporeal CO_2_ removal.

